# Estimating latent neuronal nonlinear dynamics by sequential Monte Carlo method and sparse modeling

**DOI:** 10.1007/s10827-026-00947-4

**Published:** 2026-08-04

**Authors:** Nodoka Motonishi, Toshiaki Omori

**Affiliations:** 1 Department of Electrical and Electronic Engineering, Graduate School of Engineering, Kobe University, 1–1 Rokkodai-cho, Nada-ku, Kobe, 657–8501 Hyogo Japan; 2 Center for Mathematical and Data Sciences, Kobe University, 1–1 Rokkodai-cho, Nada-ku, Kobe, 657–8501 Hyogo Japan; 3 Center of Optical Scattering Image Science, Kobe University, 1–1 Rokkodai-cho, Nada-ku, Kobe, 657–8501 Hyogo Japan; 4 Center for Life Photonic Innovation, Kobe University, 1–1 Rokkodai-cho, Nada-ku, Kobe, 657–8501 Hyogo Japan

**Keywords:** Conductance-based neuron model, Data-driven method, State space model, Sparse modeling, Bayesian inference

## Abstract

Understanding complex neuronal behavior in our brain requires accurate estimation of neuronal models from observed time-series data. In this study, we propose a data-driven sparse modeling method to estimate multi-dimensional latent variables and electrical properties while extracting essential membrane currents from partially observable time series data. First, we derive a nonlinear state space model from a conductance-based neuron model that couples membrane potential and calcium concentration including a set of candidate membrane currents. Then, we derive a sparse modeling-based expectation-maximization (EM) algorithm. In the expectation step, the expectation of the log-likelihood with Laplace prior distribution is evaluated using the posterior distribution of latent states, which is approximated by a sequential Monte Carlo (SMC) method under one-dimensional noisy observations. In the maximization step, the conductances are estimated as zero value for unnecessary currents whereas those are estimated as non-zero value for necessary currents. We show using simulation with conductance-based neuron model that the proposed method can extract the latent nonlinear neuronal dynamics from partially observable data by estimating latent variables and biophysical parameters and extracting only necessary membrane currents.

## Introduction

Estimating neuronal dynamics from observable data is important for understanding neuronal behavior (Chialvo, 2010; Shine et al., 2021). Although observation methods have been advanced for intracellular and extracellular recordings, observation provides partial and noisy information compared with neural systems (Adam et al., 2019; Piatkevich et al., 2019; Vogt, 2019); we can observe one-dimensional time-series data for individual neurons while neuronal dynamics is complex and multi-dimensional (Gerstner et al., 2014; Koch, 1999).

Data-driven approaches have been extensively investigated to estimate nonlinear dynamics (Geweke & Tanizaki, 2001; Inoue et al., 2021; Omori & Hukushima, 2016; Omori et al., 2016; Otsuka & Omori, 2019; Paninski, 2010; Wang & Beaumont, 2004). In computational neuroscience, various methods have been proposed to estimate neuronal dynamics from observable data. In particular, data-driven methods for conductance-based neuron models have been developed to estimate biologically plausible neuronal dynamics. For example, some methods estimate neuronal latent variables by assuming that biophysical parameters are known (Paninski, 2010). Other studies used statistical methods to estimate both latent variables and parameters that govern membrane potential dynamics in realistic neuron models, such as conductance-based neuron models (Inoue et al., 2021; Omori & Hukushima, 2016). However, these previous studies assumed that the type of membrane currents was known, whereas the type of membrane currents governing neuronal dynamics is unknown under many realistic situations.

Another practical issue in data-driven approaches in neuroscience is the existence of large numbers of membrane currents. In previous studies, exhaustive combination searches were conducted to reveal the necessary membrane currents from candidates by using high-performance computing (Prinz et al., 2004; Taylor et al., 2009). However, in many situations, computational explosion limits such exhaustive approaches. Thus, it is important to develop methods that extract only necessary membrane currents from partial observation data while estimating multi-dimensional latent variables and biophysical parameters from partial observation.

In this study, we propose a data-driven method for estimating neuronal dynamics in conductance-based neuron models from one-dimensional noisy observations by means of sequential Monte Carlo (SMC) method and a sparse modeling approach. The SMC method is a data-driven approach for estimating nonlinear dynamics from partial noisy observation, whereas the sparse modeling approach is a data-driven method for extracting only necessary elements from candidates. By integrating these methods alongside the expectation-maximization (EM) algorithm, we develop a data-driven method for estimating neuronal dynamics under realistic situations. First, we formulate a state-space model of the conductance-based neuron model under partial observation. Next, in order to estimate multi-dimensional latent variables from partial observation data, we derive the SMC method (Fitzgerald, 2001; Luengo et al., 2020) for the proposed state-space model under a partial observation process. Furthermore, we derive the EM algorithm (Beck & Teboulle, 2009; Moon, 1996; Otsuka & Omori, 2019) with sparse modeling for estimating biophysical parameters and extracting only necessary membrane currents simultaneously. We evaluate the proposed methods using a conductance-based model and demonstrate accurate estimation and robust current selection under one-dimensional observation data.

This paper is organized as follows. In Section 2, we propose a data-driven method for estimating multi-dimensional latent neuronal dynamics from partially observable data. In Section 3, we verify the effectiveness of the proposed method by numerical simulations with conductance-based neuron model. The concluding remarks are given in Section 4.

## Proposed method

We propose a data-driven framework for estimating multi-dimensional neuronal latent variables from a one-dimensional noisy observation based on sparse estimation. This section is organized into three parts. First, we introduce the neuron model and formulate a state space model that includes the membrane potential with many candidates of calcium currents and intracellular calcium ion concentration. Second, we derive a sequential Monte Carlo method that estimates the latent variables from the observation model of the state space model. Third, we derive an extended EM algorithm that identifies an appropriate subset of candidate currents sparsely and estimates the model parameters.

### State space model for nonlinear dynamics of conductance-based neuron model

Here, we derive a state-space model from a conductance-based neuron model that includes both membrane potential and intracellular calcium dynamics (Hodgkin & Huxley, 1952; Izhikevich, 2003; Mainen & Sejnowski, 1996; Petousakis et al., 2023).

Here we assume that membrane potential *V* obeys a conductance-based neuron model. The dynamics of membrane potential *V* is expressed by the following differential equation:

1 $$\begin{aligned} C_{\textrm{m}} \frac{dV}{dt} = - g_{\textrm{L}}(V-E_{\textrm{L}}) - I_{\textrm{ion}} + I_{\textrm{ext}}+\xi _{V}(t) . \end{aligned}$$

Here, $C_{\textrm{m}}$ denotes the membrane capacitance. The first term expresses a leak current with leak conductance $g_{\textrm{L}}$ and reversal potential $E_{\textrm{L}}$. $I_{\textrm{ion}}$ and $I_{\textrm{ext}}$ express a set of membrane currents and an external input current, respectively. $\xi _V(t)$ is a system noise for the membrane potential.

We assume a situation where there exists a set of candidate membrane currents as follows:

2 $$\begin{aligned} I_{\textrm{ion}}= \sum _{j=1}^J g_{\textrm{ion},j} f_j(V,[{\textrm{Ca}}^{2+}]) m_{j}^{p_j}(t)h_{j}^{q_j}(t)\bigl (V(t)-E_j\bigr ) \end{aligned}$$

where $g_{\textrm{ion},j}$ is a membrane conductance of *j*-th membrane current, $f_j(V,[{\textrm{Ca}}^{2+}])$ is a function of membrane potential and calcium concentration. $m_{j}$ and $h_{j}$ are gating variables for activation and inactivation, respectively, where $p_j$ and $q_j$ are the corresponding exponents with non-negative integers. $E_j$ is a reversal potential of *j**-*th membrane current.

In Eq. (2), only a part of candidate membrane currents is expected to contribute to the neuronal dynamics. We can represent the contribution of the *j*-th candidate by its maximum conductance $g_{\textrm{ion},j}$: necessary currents for latent neural dynamics have $g_{\textrm{ion},j}\ne 0$, whereas unnecessary currents should be set to $g_{\textrm{ion},j}= 0$. Therefore, selective current estimation requires shrinking small $g_{\textrm{ion},j}$ values to zero and keeping only the truly contributing components.

The gating variables $m_{j}$ and $h_{j}$ are governed by the first-order kinetics:

3 $$\begin{aligned} \frac{d m_{j} }{dt}= & \alpha _{m,j}(V)(1-m_{j})-\beta _{m,j}(V)m_{j}+\xi _{m_{j}}(t)\end{aligned}$$

4 $$\begin{aligned} \frac{d h_{j} }{dt}= & \alpha _{h,j}(V)(1-h_{j})-\beta _{h,j}(V)h_{j}+\xi _{h_{j}}(t) \end{aligned}$$

Here, $\alpha _{m,j}(V)$, $\alpha _{h,j}(V)$, $\beta _{m,j}(V)$, and $\beta _{h,j}(V)$ are nonlinear functions of membrane potential (more generally, these functions may depend on calcium concentration as well). $\xi _{m_{j}}(t)$ and $\xi _{h_{j}}(t)$ are system noises for the gating variables.

Furthermore, the dynamics of the intracellular calcium ion concentration $[\textrm{Ca}^{2+}]$ is described as follows:

5 $$\begin{aligned} \frac{d[\textrm{Ca}^{2+}]}{dt} = -\alpha _{\textrm{Ca}} I_{\textrm{Ca}} - \frac{[\textrm{Ca}^{2+}]-[\textrm{Ca}^{2+}]_{\infty }}{\tau _{\textrm{Ca}}} +\xi _{\textrm{Ca}}(t). \end{aligned}$$

The first term represents calcium influx driven by $I_{\textrm{Ca}}$, while the second term represents a decay process. Here, $\alpha _{\textrm{Ca}}$ and $\tau _{\textrm{Ca}}$ are constants, and $\xi _{\textrm{Ca}}(t)$ is a system noise for the calcium concentration.

**Fig. 1:**
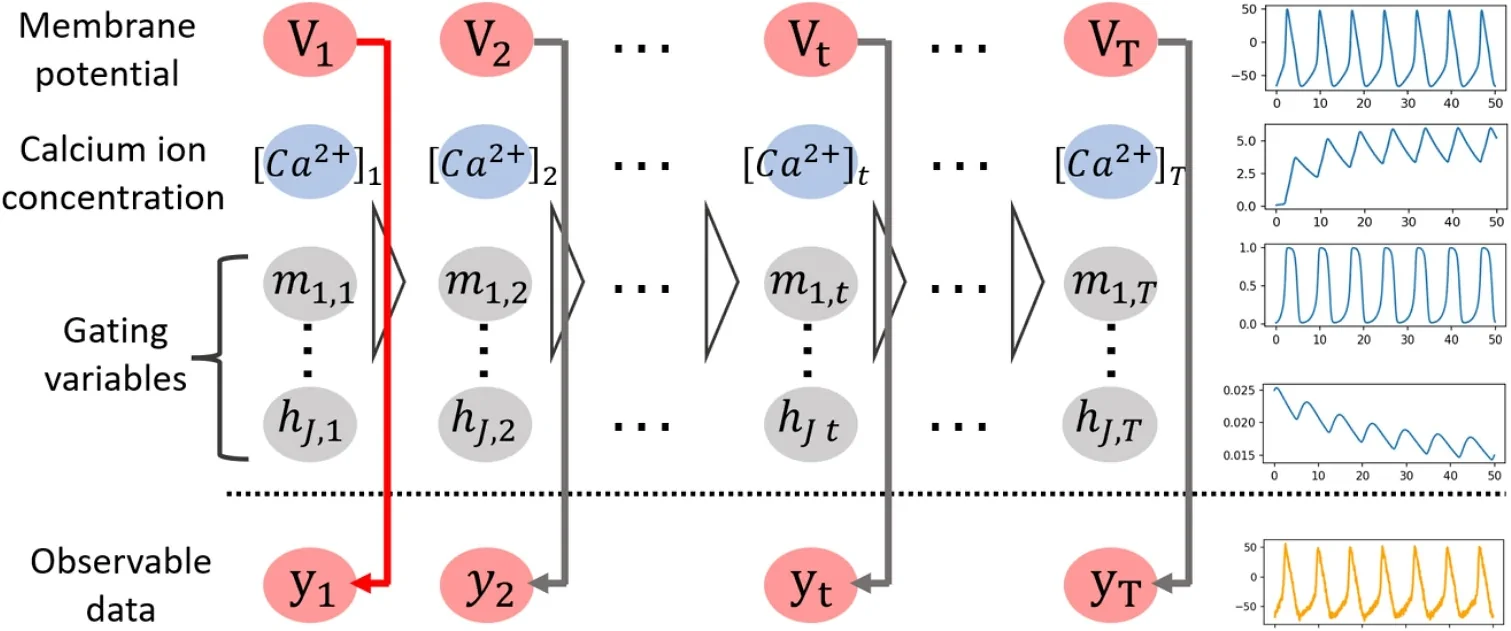
Graphical model of a state-space model in the proposed method. The system model $p(X_{t+1}|X_t)$ describes the time evolution of multi-dimensional latent variables $X_t= [V_t, [\textrm{Ca}^{2+}]_t,\{m_{j,t}\},\{h_{j,t}\}]$. On the other hand, the observation model $p(y_t|X_t)$ describes a partial observation process where only one-dimensional noisy time-series data $y_t$ can be observed

Next, we formulate a state space model. Figure 1 shows a graphical model of the state space model of the proposed method. The state space model consists of two models: a system model that describes how the latent variables transit over time, and an observation model that describes how the observed data are obtained from the latent variables.

Here, we derive the system model. We consider multi-dimensional latent variables $X_t$ for neural dynamics as follows:

6 $$\begin{aligned} X_t = \bigl [ V_t,\, [\textrm{Ca}^{2+}]_t,\, M_{t},\, H_{t}\, \bigr ]. \end{aligned}$$

Here, $M_{t}$ and $H_{t}$ are sets of gating variables for activation and inactivations, respectively as follows: $M_{t} = [m_{1,t}, \cdots , m_{J,t}]$ and $H_{t} = [h_{1,t}, \cdots , h_{J,t}]$.

By discretizing the neuron model in Eqs. (1)–(5) with respect to time by using the Euler–Maruyama approximation scheme, the state transition model is derived as follows:

7 $$\begin{aligned} X_{t+1} = f(X_t,\theta ) + \epsilon _t, \qquad \epsilon _t \sim \mathcal {N}(0,\Sigma _{\textrm{sys}}), \end{aligned}$$

where *f(· )* denotes the transition equation of the latent variables. The system model is expressed as a probabilistic model $p(X_{t+1}|X_{t},\theta)$ as the following expression:

8 $$\begin{aligned} p(X_{t+1}|X_{t}{,\theta }) \propto \exp \left( -\frac{1}{2}(X_{t+1}-f(X_{t},\theta))^\top \Sigma_{\mathrm{sys}}^{-1} (X_{t+1}-f(X_{t},\theta)) \right) \end{aligned}$$

Next we derive the observation model. Here we assume only noisy membrane potential can be observable as follows:

9 $$\begin{aligned} y_t = g(V_t) + \eta _t, \qquad \eta _t \sim \mathcal {N}(0,\sigma _{\textrm{obs}}^2). \end{aligned}$$

where *θ * is a set of parameters expressed by $\theta =[g_{\textrm{ion},1},g_{\textrm{ion},2},\cdots ,g_{\textrm{ion},J}]$, and *g(· )* is a function of membrane potential. The observation model is expressed as a probabilistic model $p(y_{t}|X_{t})$ as the following expression:

10 $$\begin{aligned} p(y_{t}|X_{t}) = \frac{1}{\sqrt{2 \pi \sigma_{\mathrm{obs}}^2}} \exp \left( - \frac{\left( y_{t}-g(V_{t}) \right)^2}{2\sigma_{\mathrm{obs}}^2} \right) \end{aligned}$$

The goal of this study is to derive a data-driven estimation method to estimate the latent state trajectory $X_{1:T}$ and the parameters $\theta =[g_{\textrm{ion},1},g_{\textrm{ion},2},\cdots ,g_{\textrm{ion},J}]^\top$ while extracting only necessary membrane currents, given the one-dimensional observation data $y_{1:T}$. Note that the parameter vector $\theta =[g_{\textrm{ion},1},g_{\textrm{ion},2},\cdots ,g_{\textrm{ion},J}]^\top$ is assumed to be constant over time. Hereafter, for notational simplicity, we also use $g_j$ to denote $g_{\textrm{ion},j}\;(j=1,\cdots,J)$ , and hence express the parameter vector as $\theta =[g_{1},g_{2},\cdots ,g_{J}]^\top$.

**Fig. 2:**
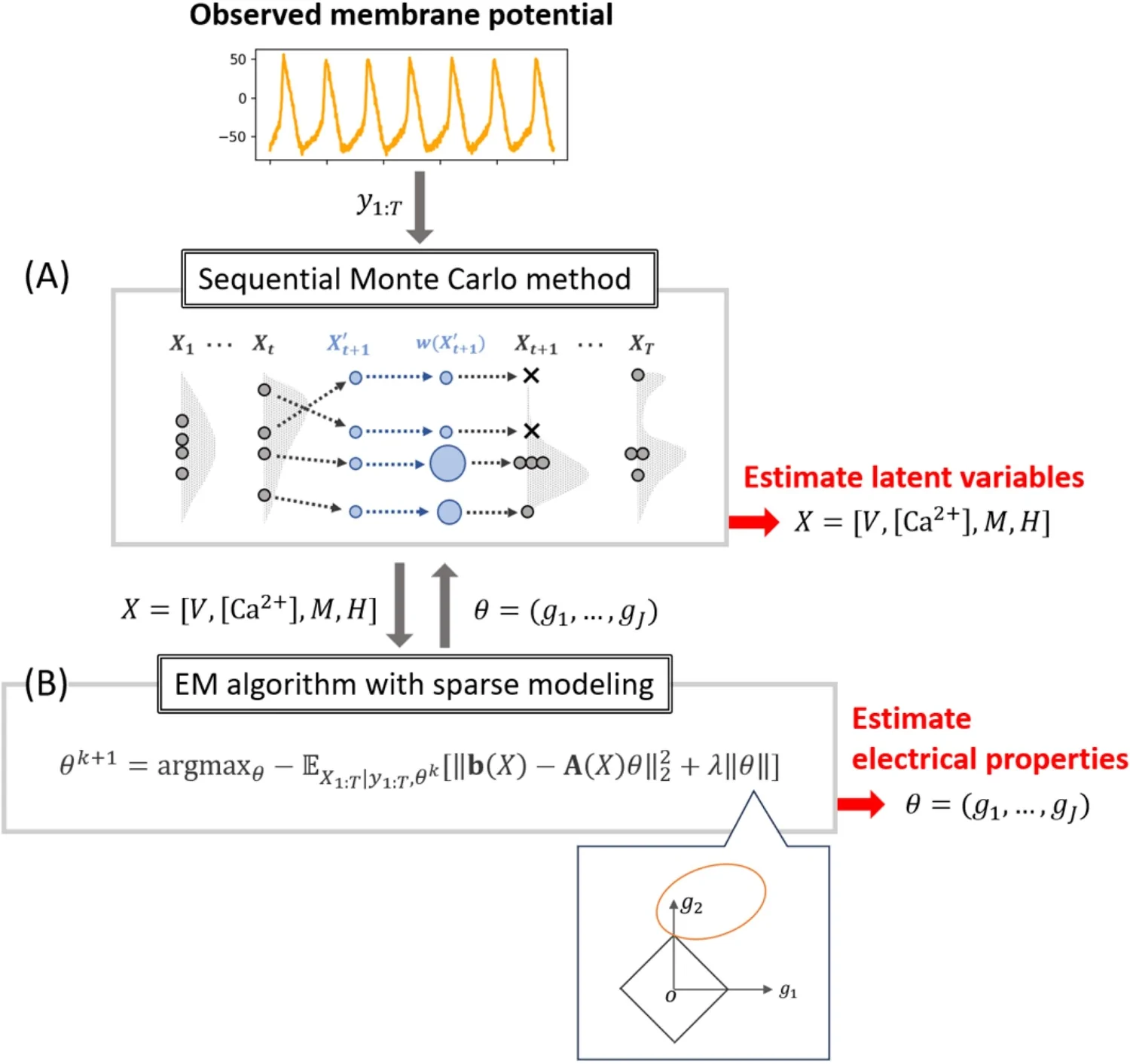
Schematic diagram of the proposed method. We estimate multi-dimensional latent variables $X_t$ by sequential Monte Carlo method whereas estimation of electrical properties *θ * and extraction of only necessary membrane currents are conducted by EM algorithm with sparse modeling

### Estimation of neuronal latent variables by sequential Monte Carlo method

Here, we describe the method for estimating the multi-dimensional latent state $X_t$ from the partially observed one-dimensional data $y_t$ using the sequential Monte Carlo method (SMC) [Fig. 2 (A)]. The target distribution is approximated by multiple particles, each comprising a sampled state vector and an associated likelihood. Note that the sequential Monte Carlo method is conducted for constant value of parameters $\theta =[g_{\textrm{ion},1},g_{\textrm{ion},2},\cdots ,g_{\textrm{ion},J}]^\top$.

First, we consider a predictive distribution $p(X_{t+1}|y_{1:t}{,\theta })$, which expresses the probability of latent variable $X_{t+1}$ at time step *t+1* given observable time series data $y_{1:t}$ up to the previous time step *t*. The predictive distribution $p(X_{t+1}|y_{1:t}{,\theta })$ is expressed by a system model $p(X_{t+1}|X_{t}{,\theta })$ as follows:

11 $$\begin{aligned} p(X_{t+1}|y_{1:t}{,\theta }) = \int p(X_{t+1}|X_{t}{,\theta }) p(X_{t}|y_{1:t}{,\theta }) dX_{t} \end{aligned}$$

where $p(X_{t}|y_{1:t}{,\theta })$ is a filtering distribution, which expresses the probability of latent variable $X_{t}$ at time step *t* given observable time series data $y_{1:t}$ up to the same time step *t*. Note that the predictive distribution $p(X_{t+1}|y_{1:t}{,\theta })$ at time *t+1* is derived by the filtering distribution $p(X_{t}|y_{1:t}{,\theta })$ at time *t*.

On the other hand, the filtering distribution $p(X_{t}|y_{1:t}{,\theta })$ is expressed by observation model $p(y_{t}|X_{t})$ as follows:

12 $$\begin{aligned} p(X_{t}|y_{1:t}{,\theta }) = \frac{p(y_{t}|X_{t}) p(X_{t}|y_{1:t-1}{,\theta })}{ \int p(y_{t}|X_{t}) p(X_{t}|y_{1:t-1}{,\theta }) dX_{t}} \end{aligned}$$

Note that the filtering distribution $p(X_{t}|y_{1:t}{,\theta })$ at time *t* is derived by the predictive distribution $p(X_{t}|y_{1:t-1}{,\theta })$ at time *t* given observable time series data $y_{1:t-1}$ up to the preceding time step *t-1*. Therefore, the multi-dimensional latent variables $X_{t}$ are estimated sequentially by conducting prediction and filtering with the state space model with system model and observation model derived in the previous subsection.

Here, we conduct the prediction and filtering by using a set of particles with three steps: one-step ahead prediction, likelihood calculation, and resampling.

First, in the one-step ahead prediction stage, each particle $X_{t}^{(i)}$ at time step *t* is propagated to $X_{t+1}^{(i)}$ at the next time step *t+1* according to the transition model *f(· )* and system model $p(X_{t+1}|X_t{,\theta })$ shown in Eqs. (7) and (8).

Second, in the likelihood calculation stage, we compute the likelihood $w_{t+1}^{(i)}$ for each particle as follows:

13 $$\begin{aligned} w_{t+1}^{(i)} \propto p\!\left( y_{t+1}\mid X_{t+1}^{(i)} \right) , \end{aligned}$$

where we use the observation model $p(y_{t+1}|X_{t+1})$ [Eq. (10)] and *i* denotes the particle number. Then the calculated weights $w_{t+1}^{(i)}$ are normalized as follows:

14 $$\begin{aligned} \tilde{w}_{t+1}^{(i)} = \frac{w_{t+1}^{(i)}}{\sum _{j=1}^{N} w_{t+1}^{(j)}}. \end{aligned}$$

Third, in the resampling stage, we resample particles according to the normalized weights to avoid weight degeneracy as follows:

15 $$\begin{aligned} \tilde{X}_{t+1}^{(i)} = X_{t+1}^{(a_i)}, \qquad \Pr (a_i=j)=\tilde{w}_{t+1}^{(j)}. \end{aligned}$$

By repeating these three steps for *t=1,… ,T-1*, we approximate the filtering distribution $p(X_t \mid y_{1:t}, \theta )$. By taking representative values, we obtain the estimated multi-dimensional latent variables $X_{1:T}$ from the one-dimensional observation data $y_{1:T}$.

### Sparse estimation of biophysical parameters by adapted EM algorithm

In this section, we derive a data-driven algorithm with sparse modeling method in order to extract only necessary membrane currents [Fig. 2 (B)]. We derive the expectation-maximization (EM) algorithm with a prior distribution of biophysical parameters. The EM algorithm consists of two steps: E-step and M-step.

In the E-step, using the posterior distribution approximated by sequential Monte Carlo (SMC), we evaluate the *Q* function, which denotes the expected complete-data log-likelihood as follows:

16 $$\begin{aligned} \begin{aligned} Q(\theta \mid \theta ^{(k)}) = \mathbb {E}_{X_{1:T} \mid y_{1:T}, \theta ^{(k)}} \left[ \log p(X_{1:T}, y_{1:T} , \theta ) \right] \end{aligned} \end{aligned}$$

where the joint probability $p(X_{1:T}, y_{1:T}, \theta )$ is expressed by probabilistic expression of state space model as follows:

17 $$\begin{aligned} p(X_{1:T}, y_{1:T}, \theta ) = \left( \prod _{t=1}^{T-1} p(X_{t+1}|X_{t},\theta ) \right) \left( \prod _{t=1}^{T} p(y_{t}|X_{t}) \right) p(\theta ) \end{aligned}$$

and $\mathbb {E}_{X_{1:T} \mid y_{1:T}, \theta ^{(k)}}[\cdot ]$ denotes an expectation over the posterior distribution with parameter at *k*-th step as follows:

18 $$\begin{aligned} \mathbb {E}_{X_{1:T} \mid y_{1:T}, \theta ^{(k)}}[q(X_{1:T})] = \int q(X_{1:T}) p(X_{1:T}|y_{1:T},\theta ^{(k)}) d X_{1:T} \end{aligned}$$

By substituting the joint probability into *Q* function, we obtain the following expression:

19 $$\begin{aligned}Q(\theta \mid \theta ^{(k)})& = \mathbb {E}_{X_{1:T} \mid y_{1:T}, \theta ^{(k)}} \Biggl [ \sum _{t=1}^{T-1} \log p(X_{t+1} \mid X_t, \theta )\\& + \sum _{t=1}^{T} \log p(y_t \mid X_t) + \log p(\theta ) \Biggr ] \end{aligned}$$

where *p(θ )* denotes a prior distribution of parameters.

**Fig. 3:**
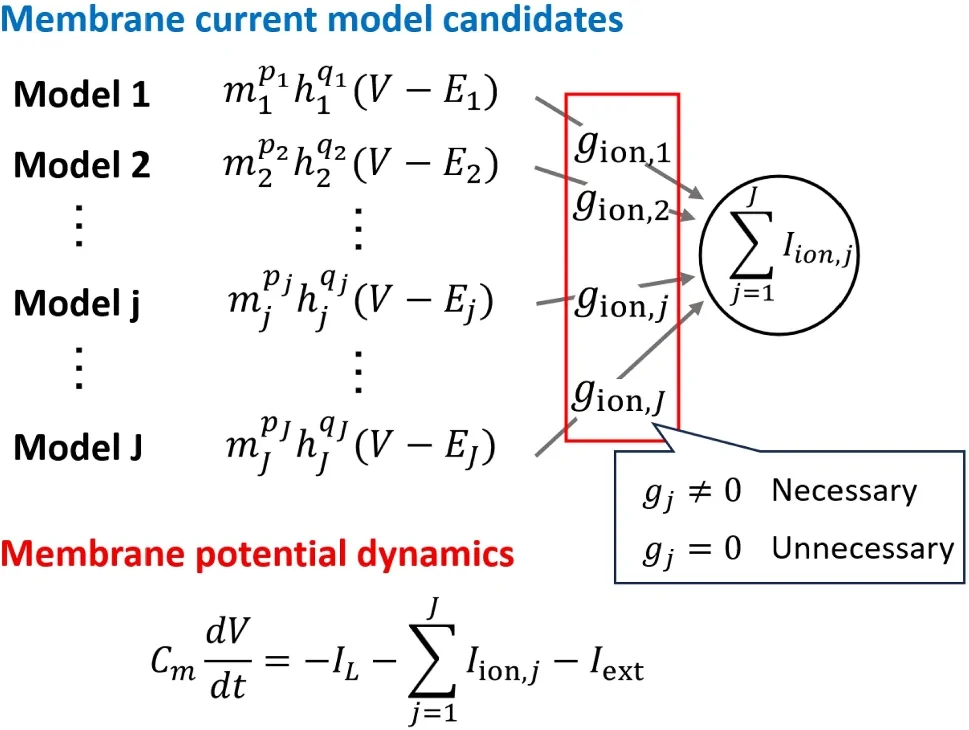
Schematic diagram of sparse modeling for neuronal dynamics. By means of sparse modeling, each element in a set of parameters $\theta =[g_{\textrm{ion},1},g_{\textrm{ion},2},\cdots ,g_{\textrm{ion},J}]^\top$ is estimated as zero value $g_{\textrm{ion},j} = 0$ for unnecessary membrane current whereas non-zero value $g_{\textrm{ion},j}\ne 0$ for necessary membrane current

Figure 3 shows a schematic diagram of sparse modeling. In order to extract only necessary membrane currents from candidates, we consider a Laplace distribution on *θ * as follows:

20 $$\begin{aligned} p(\theta ) \propto \exp (-\lambda \Vert \theta \Vert _1 ) \end{aligned}$$

where $\Vert \theta \Vert _1$ corresponds to an $L_1$ norm, and *λ * is a non-negative constant. By means of sparse estimation with the Laplace prior distribution, non-zero values are estimated for necessary membrane currents, whereas zero values are assumed for unnecessary membrane currents.

In the M step, we obtain a parameter *θ * by maximizing *Q* function with respect to parameter *θ * as follows:

21 $$\begin{aligned} \theta ^{(k+1)} = \arg \max _{\theta } Q(\theta \mid \theta ^{(k)}) \end{aligned}$$

Here, we derive the mathematical formulation of M-step by means of sparse modeling. We consider an explicit expression of the probabilistic model for the system model.

22 $$\begin{aligned} p(V_{t+1}|X_{t},\theta) \propto \exp \left[ -\frac{1}{2\sigma _V^2} \left( V_{t+1}-V_{t}+\frac{\Delta t}{C_{\textrm{m}}} \left( I_{\textrm{L},t}+I_{\textrm{ion},t}-I_{\textrm{ext},t}\right) \right) ^2 \right] \end{aligned}$$

where *Δ t* is a time width for discretization. Here, $\sigma_V$ denotes the standard deviation of the membrane-component of the system noise; thus, $\sigma _{V}^2$ is the corresponding diagonal element of the covariance matrix ${\sum}_\mathrm{sys}$. The difference at step *t* of membrane potential is expressed by the following expression:

23 $$\begin{aligned} \Delta V_t&= \frac{ V_{t+1}- V_{t}}{\Delta t} \end{aligned}$$

Here we summarize terms in Eq. (22) into two factors: the one without membrane currents and the other with membrane currents. First we express terms $b_t$ without membrane currents as follows:

24 $$\begin{aligned} b_t = C_{\textrm{m}} \Delta V_t + I_{\textrm{L},t}-I_{\textrm{ext},t} \end{aligned}$$

Next, we consider terms with membrane currents. Here we consider that there exist *J* candidates for membrane currents with maximal membrane conductances $\theta =[g_{\textrm{ion},1},\dots ,g_{\textrm{ion},J}]^\top$. The *j*-th candidate membrane current at time step *t* is expressed by the following expression:

25 $$\begin{aligned} I_{\textrm{ion},j,t}= & g_{\textrm{ion},j}\, m_{j,t}^{p_j}\,h_{j,t}^{q_j}\,( V_t-E_{j}) \nonumber \\= & g_{\textrm{ion},j} \phi _j(X_t) \end{aligned}$$

where $m_{j,t}$ and $h_{j,t}$ are gating variables with exponents $p_j$ and $q_j$, respectively. Here we put the candidate term for *j*-th components is expressed by

26 $$\begin{aligned} \phi _j(X_t) = m_{j,t}^{p_j}\,h_{j,t}^{q_j}\,( V_t-E_j) \end{aligned}$$

as one of basis functions.

By substituting Eqs. (22) into (21), we derive the update rule for estimating parameters obeying minimization problem with a reconstruction term of dynamics and an $L_1$ norm regularization as follows:

27 $$\begin{aligned} \theta ^{(k+1)}= & {\arg \max _\theta \mathbb {E}_{X_{1:T} \mid y_{1:T}, \theta ^{(k)}} \left[ \sum _{t=1}^{T-1} \log p(X_{t+1}|X_t,\theta ) + \log p(\theta )\right] } \nonumber \\= & \arg \min _\theta \mathbb {E}_{X_{1:T} \mid y_{1:T}, \theta ^{(k)}} {\left[ \left\| b - A \theta \right\| _2^2 +\lambda \Vert \theta \Vert _1 \right] } \end{aligned}$$

where *λ * is a regularization constant ($\lambda \ge 0$). The first term corresponds to the reconstruction error of the neuronal dynamics, whereas the second term originates from the Laplace prior distribution and induces sparsity. Note that maximizing the *Q* function is equivalent to minimizing the objective in Eq. (27), due to negative signs arising in the logarithms of both the system model and the prior distribution (i.e., $\log p(X_{t+1}|X_t,\theta ) \simeq -\left\| b - A \theta \right\| _2^2$ and $\log p(\theta ) \simeq -\lambda \Vert \theta \Vert _1$). Here, a matrix *A* is expressed with the following elements:

28 $$\begin{aligned} A_{t,j} = -\phi _j(X_t), \qquad t=1,\dots ,T-1,\;\; j=1,\dots ,J, \end{aligned}$$

and a vector $b=[b_1,\dots ,b_{T-1}]^\top$. Note that the sparse structure is automatically induced by the $L_1$ regularization term. No explicit thresholding operation is applied during the estimation process. Instead, the optimization based on $L_1$ regularization shrinks unnecessary conductance parameters to exactly zero, while retaining only the conductances that contribute to explaining the observed membrane potential dynamics.

Bottom figure in Fig. 3 shows a mechanism of sparse modeling. For substantial understanding, a two dimensional case $\theta =[g_1, g_2]^\top$ is shown. Since first and second terms in Eq. (27) correspond to an ellipse and a diamond with origin center, respectively, a sparse solution is obtained; a zero element is estimated as $\theta =[0,g_2]^\top$. Therefore, for general high-dimensional case, in terms of regularization based on the $L_1$ norm, necessary membrane currents are estimated as $g_{\textrm{ion},j} \ne 0$, whereas unnecessary membrane currents are estimated as $g_{\textrm{ion},j} = 0$. Therefore, the number of active membrane currents is determined automatically by the optimization procedure and does not need to be specified a priori.

By repeating the sequential Monte Carlo method and the EM algorithm with sparse modeling, we simultaneously estimate the latent trajectory $X_{1:T}$ and the parameters *θ *. The sparsity regularization pushes unnecessary candidate currents to zero, so only necessary currents are selectively estimated. In summary, we have derived the EM-based method where the expectation with respect to multi-dimensional latent variables $X_t$ is conducted by using posterior distribution obtained by the sequential Monte Carlo method, and the M-step enforces sparsity for parameters *θ * to select only necessary membrane currents from candidates. The number of active ion channels does not need to be specified in advance. All candidate conductances are included in the estimation, and sparse channel selection is achieved automatically through $L_1$ regularization. No explicit thresholding procedure is employed.

## Results

### Settings

To evaluate the proposed method, we used a biologically reasonable conductance-based neuron model (De Schutter & Bower, 1994; Destexhe et al., 1998; Destexhe & Sejnowski, 2001; Pospischil et al., 2008; Wang et al., 1991). We assume that one-dimensional noisy time-series data of the membrane potential is observed. The latent variables *X* include the membrane potential, calcium ion concentration, and the gating variables of each current model. In this study, we focus on calcium currents, for which many candidates have been proposed. We therefore prepare *J* candidate calcium currents. As true neuronal model, we consider the conductance neuron model with both membrane dynamics and calcium dynamics as follows:

29 $$\begin{aligned} C_\textrm{m} \frac{dV}{dt} = - I_{\textrm{L}} - I_{\textrm{T}} - I_{\textrm{Ca}} - I_{\textrm{Na}}-I_{\textrm{K}}-I_{\textrm{KCa}}-I_{\textrm{A}} +I_{\textrm{ext}} \end{aligned}$$

where true dynamics includes two types of calcium currents: the low-threshold T-type calcium current $I_{\textrm{T}}$ and the high-voltage-activated calcium current $I_{\textrm{Ca}}$ (Destexhe et al., 1998; Destexhe & Sejnowski, 2001). In addition, the model contains standard sodium and potassium membrane currents, namely, the sodium current $I_{\textrm{Na}}$, the delayed rectifier potassium current $I_{\textrm{K}}$, the calcium-dependent potassium current $I_{\textrm{KCa}}$ and the A-type potassium current $I_{\textrm{A}}$ (Connor & Stevens, 1971; Feng, 2004; Johnston & Wu, 1995).

We estimate the high-dimensional latent variables $X = [V, [\textrm{Ca}^{2+}], m_1 , ... , m_J, h_1, ..., h_J, m, h, n, m_{\textrm{A}}, h_{\textrm{A}}]$ with membrane potential *V*, calcium concentration $[\textrm{Ca}^{2+}]$, gating variables for calcium currents $M=[m_1 , ... , m_J]$ and $H=[h_1, ..., h_J]$ and sodium and potassium currents $m, h, n, m_{\textrm{A}}, h_{\textrm{A}}$, as well as the maximal conductances $g_j$ of the candidate calcium currents $I_j = g_j\, f(V, [\textrm{Ca}^{2+}])\, m_j^{p_j} h_j^{q_j} (V - E_{\textrm{Ca}})$ by means of the proposed method. If a candidate current is necessary to explain the dynamics, the method estimates $g_j \ne 0$. Otherwise, it shrinks $g_j$ to zero. In the proposed method, latent variables and biophysical parameters are estimated simultaneously using a single observed time-series, although multiple time-series datasets can be incorporated in principle. The regularization constant *λ * in Eq. (27) is automatically determined by 5-fold cross-validation. The observed time-series data are divided into five folds: biophysical parameters are estimated using four folds, and the remaining fold is used for validation. The value of *λ * that minimizes the validation error is selected.

We also conduct the estimation of latent variables and biophysical parameters by means of the framework without sparse modeling for comparison. In this comparison framework, the Laplace prior distribution on the conductance parameters is removed and no alternative prior distribution is introduced. Since previous studies on latent neuronal dynamics estimation from partial observations generally assumed known membrane current types, this framework serves as a baseline for evaluating the effectiveness of sparse modeling under the same estimation setting.

### Estimation of multi-dimensional latent variables

**Fig. 4:**
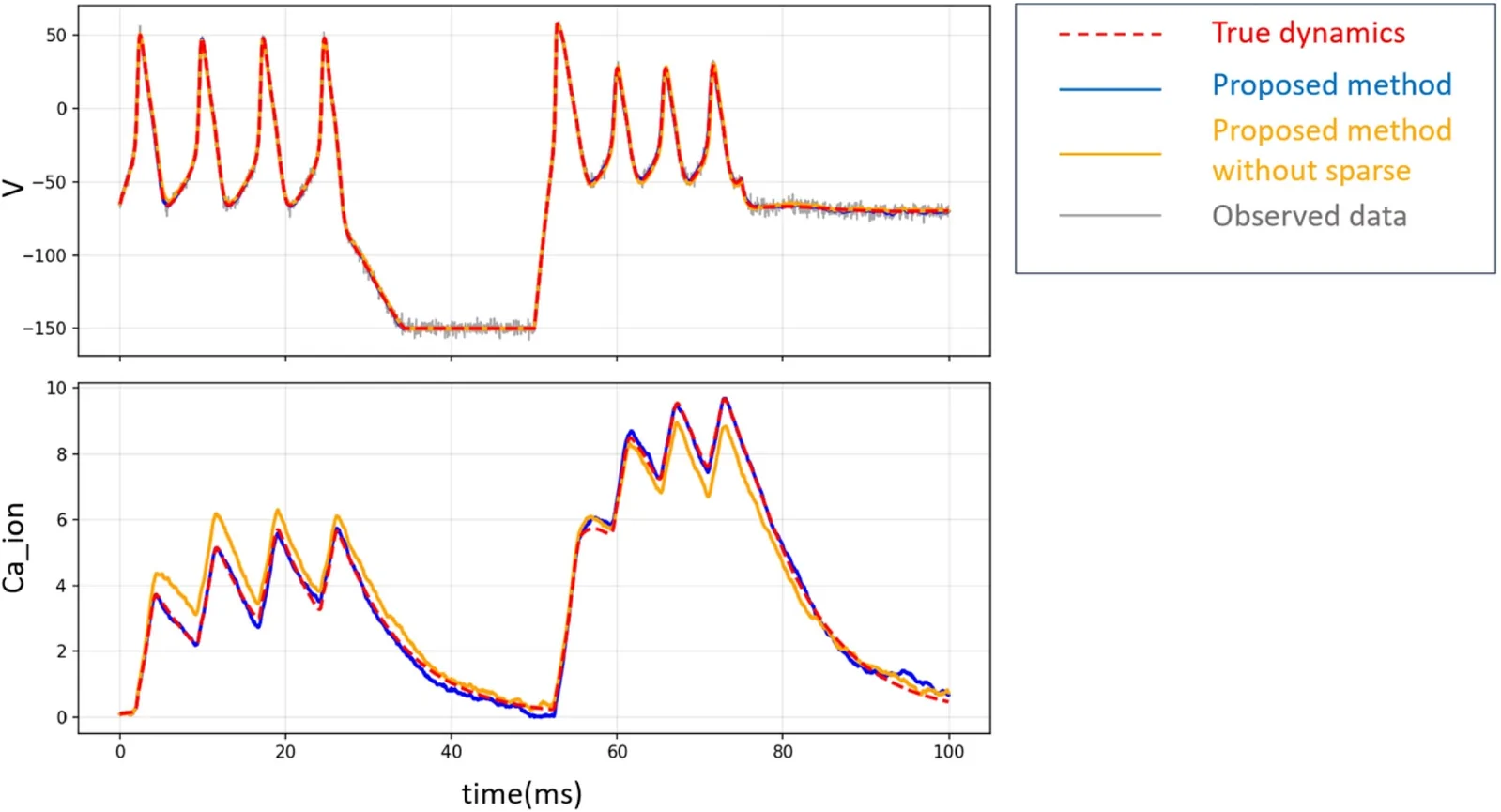
Estimation of multi-dimensional latent variables from partially observable, one dimensional noisy membrane potential observations. Estimated membrane potential *V* and calcium concentration $[\textrm{Ca}^{2+}]$ are shown. The red dashed line represents the true latent variables, the blue solid line shows the latent variables estimated by the proposed method, the orange solid line shows the latent variables estimated by the proposed method without sparse modeling, and lastly, the gray line means the observed noisy membrane potential $y_t$

Here, we estimate the latent variables *X* from one-dimensional noisy membrane potential observations only. Figure 4 shows the estimation results of the membrane potential *V* and the calcium ion concentration $[\textrm{Ca}^{2+}]$ obtained by the sequential Monte Carlo step in the proposed method, using the estimates at the final iteration.

The upper panel of Fig. 4 shows the estimation of the observed membrane potential. Both the proposed method (blue line) and the proposed method without sparse modeling (orange line) effectively suppress observation noise and closely follow the true dynamics (red line). As shown in Table 1, the proposed method achieves a smaller estimation error between true and estimated membrane potential.

On the other hand, the lower panel of Fig. 4 shows the estimation of the unobserved calcium dynamics. The proposed method without sparse method fails to track the spike of the calcium dynamics, whereas the proposed method succeeds in following the true dynamics. This difference is also reflected in Table 1, where the proposed method achieves a smaller error in reconstructing $[\textrm{Ca}^{2+}]$ compared with the one without sparse modeling.

These results indicate that, even when only the membrane potential is observed, the proposed method can accurately estimate multi-dimensional latent variables. Moreover, incorporating sparse modeling improves the estimation of the latent variables compared with the framework without sparsity.

### Selective estimation of membrane current models

Here, we estimate the electrical parameters and select the necessary current models from *J=16* candidate calcium currents. We set the initial parameters as $\theta ^{(0)}=\left[ g_1^{(0)}, \dots , g_{16}^{(0)}\right]^\top =[0.2, \dots ,\, 0.2]^\top,$ and assume observation noise with standard deviation $\sigma _{\textrm{obs}}=2.7$ for the membrane potential. As the final parameter estimate, we take the average over the last 50 iterations after convergence.

**Fig. 5:**
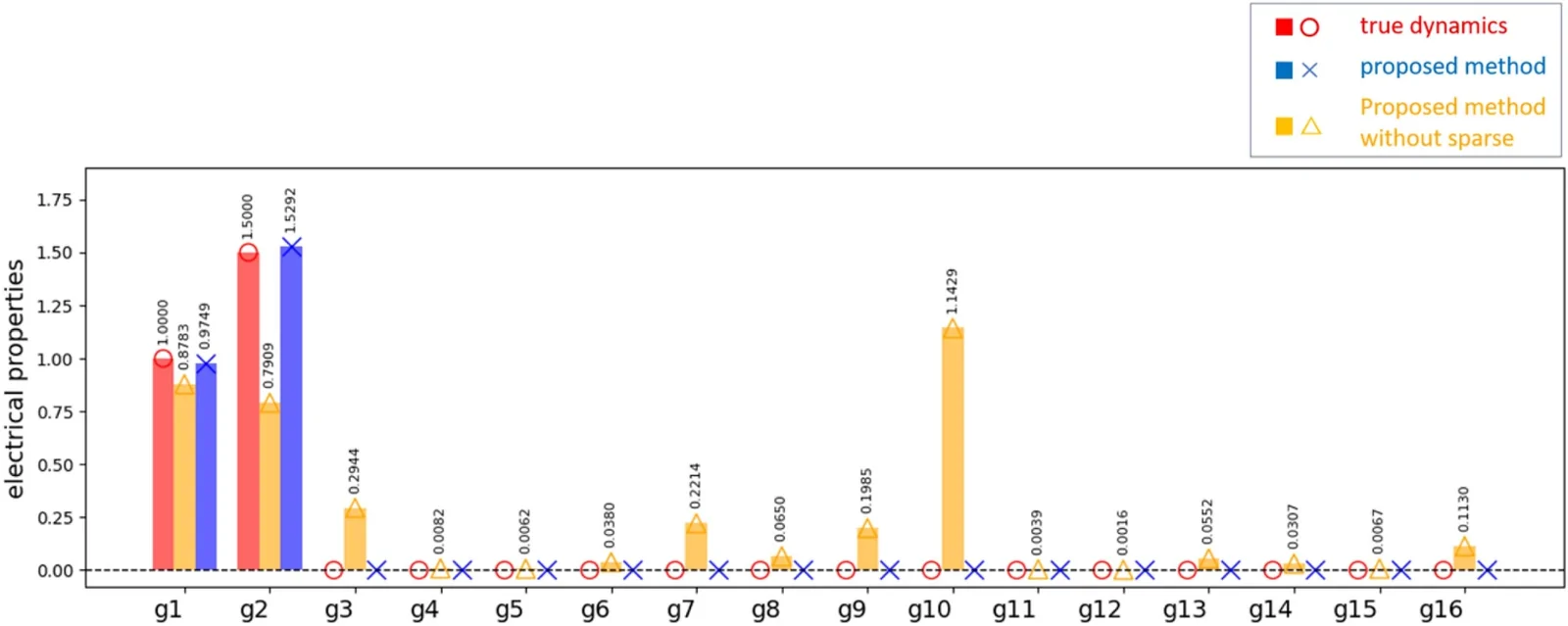
Estimated maximal conductances $g_j$ for *J=16* candidate calcium currents from one-dimensional noisy membrane potential observations. The red bars denote the true maximal conductances, the blue bars indicate the estimates obtained by the proposed method, and the orange bars show the results obtained by the proposed method without sparse modeling

Figure 5 shows the conductance estimates obtained from membrane potential observations. The red bars indicate the true electrical properties, the blue bars denote the proposed method with sparse regularization, and the orange markers show the proposed method without sparse regularization.

As shown in Fig. 5, the proposed method with sparse modeling successfully extracts only necessary membrane currents. The conductances corresponding to the truly necessary currents ($g_1$ and $g_2$) are estimated as non-zero value and the estimated values ($g_1=0.9749$ and $g_2=1.5292$) are quite close to the true values ($g_1=1.0$ and $g_2=1.5$). In addition, by means of the proposed method with sparse modeling, the conductances corresponding to the truly unnecessary currents ($g_3$, $g_4$, $\cdots , g_{16}$) are estimated as zero values; the proposed method properly shrinks the membrane conductances of unnecessary candidates toward zero and removes them.

On the other hand, the proposed method without sparse modeling shows severe results in the selection of membrane currents. The proposed method without sparse modeling estimates the conductances corresponding to the truly necessary currents ($g_1$ and $g_2$) as non-zero. However, the estimated non-zero values ($g_1=0.8783$ and $g_2=0.7909$) are relatively different from the true ones ($g_1=1.0$ and $g_2=1.5$). Moreover, the proposed method without sparse modeling also assigns non-zero values to many conductances that should be zero ($g_3$ to $g_{16}$). This indicates overfitting and a failure of membrane current selection when many candidate currents are included. In other words, unnecessary current models remain active, and the estimated model becomes overly complex as the number of candidates increases.

**Fig. 6:**
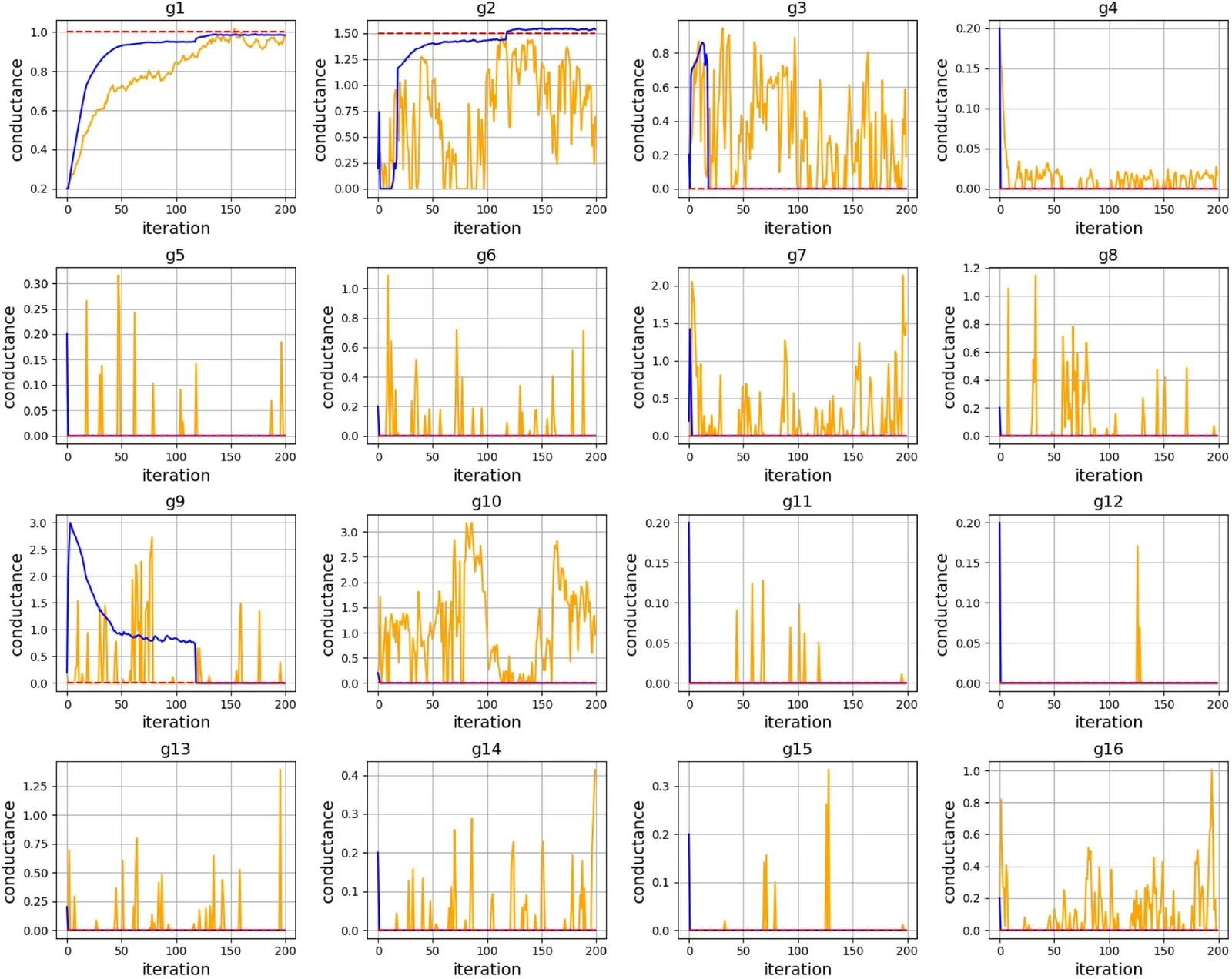
Convergence tracks of the conductance estimates for *J=16* candidate calcium currents from one-dimensional noisy membrane potential observations. The red dashed lines denote the maximal conductances, the blue lines show the iterative updates of the proposed sparse method, and the orange lines show those of the proposed method without sparse modeling

Next, Fig. 6 shows the transient behavior of the estimation results of the electrical properties. Figure 6 plots the iterative updates of conductances based on membrane potential observations, where the red dashed lines denote the true electrical properties, the blue lines denote the proposed method, and the orange lines denote the proposed method without sparse modeling. In the proposed method without sparse modeling (orange), although $g_1$ appears to converge around iteration *∼ 150*, the estimates of the conductances remain unstable even after this point. With many irrelevant candidates, the method struggles to separate current contributions, and some effects are wrongly attributed to other currents.

In contrast, for the proposed method (blue), the conductances of the truly necessary currents, $g_1$ and $g_2$, converge to the true values around iteration *∼ 120*, while the conductances of the other candidates are set to 0. Moreover, the estimates remain stable after convergence. These results suggest that sparse regularization suppresses weakly contributing candidates and enables selection of only the truly contributing currents.

Overall, the proposed method can estimate conductance parameters while selecting current models from many candidates, even under partial observation, where only the membrane potential is available.

To further evaluate robustness against observation noise, we applied the proposed method to 12 independently generated observation sequences with different observation-noise realizations. Table 2 summarizes the estimated conductances. The proposed method consistently identified the active currents and accurately estimated their conductances across all runs, while the remaining conductances were correctly suppressed to zero.

**Table 1:** MSE of the estimated latent variables using the proposed method and the proposed method without sparse modeling for membrane potential and calcium concentration

	MSE(*V*)	MSE([$\textrm{Ca}^{2+}]$)
Proposed method	0.723886	0.028215
Proposed method without sparse modeling	3.400895	0.249483

**Table 2:** Robustness of the proposed method evaluated using 12 independently generated observation sequences with different observation-noise realizations. Mean and standard deviation (SD) of the estimated maximal conductances are shown. The true model contains only two active calcium currents ($g_1=1.0$ and $g_2=1.5$), whereas the remaining conductances should be estimated as zero

Conductance	True value	Mean estimate	SD
$g_1$	1.0	0.999	0.022
$g_2$	1.5	1.500	0.050
$g_3$–$g_{16}$	0.0	0.000	0.000

### Evaluation of generalization performance

**Fig. 7:**
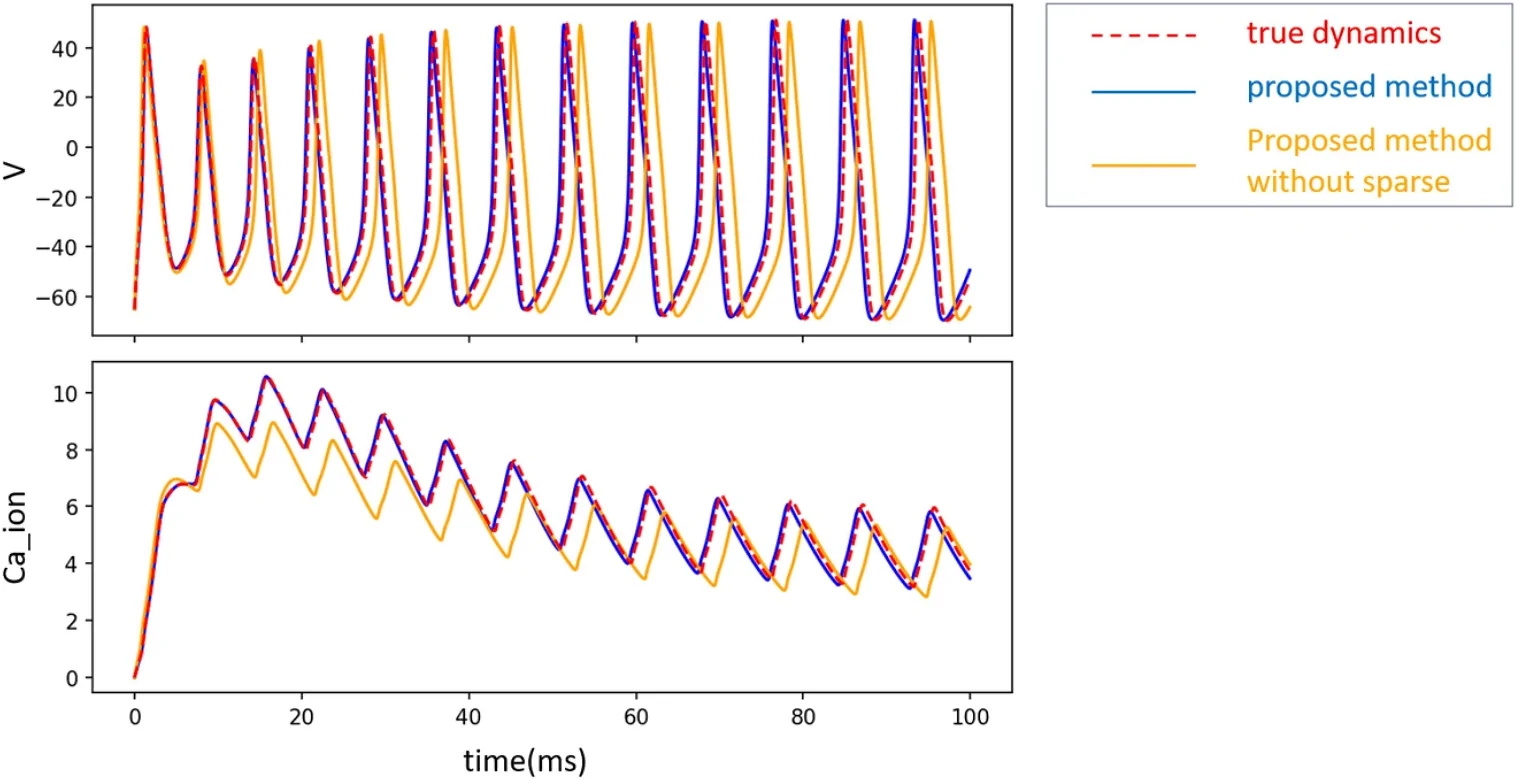
Generalization performance under an input current different from that used during training. The red dashed line denotes the true dynamics, the blue solid line indicates the proposed method, and the orange solid line indicates the proposed method without sparse modeling

Here, we compare the generalization performance of the proposed method with that of the proposed method without sparse modeling. Using the estimated conductances obtained in Fig. 5, we reconstruct the latent variables under input current different from the input current $I_{\textrm{ext}}$ used during training. The results are shown in Fig. 7. The red dashed line denotes the true dynamics, the blue solid line denotes the generalized track using the electrical properties estimated by the proposed method, and the orange solid line denotes that by the proposed method without sparse modeling.

In the upper panel (membrane potential), the proposed method without sparse modeling shows an inaccurate trajectory in spike amplitude from the beginning, and its spike timing gradually drifts over time. In contrast, the proposed method closely tracks the true dynamics. This difference is even more emphasized in the lower panel (calcium concentration), where the proposed method reproduces the calcium dynamics more accurately.

These results suggest that the proposed method estimates the electrical properties $\theta = [g_1, \ldots , g_{16}]^\top$ with smaller error and performs appropriate model selection, leading to better generalization performance.

### Effectiveness of proposed method under different sparsity levels

To evaluate the effectiveness of the proposed method under different sparsity levels, we investigate whether the proposed method can extract only necessary membrane currents by changing the number of candidate currents *J* in the true models. Although the true model contains only two currents, $I_1$ and $I_2$, we prepared candidate sets with *J=2, 4, 8, 12, 16*, and estimated the maximal conductances $g_j$. The estimated conductances $g_j$ are shown in Table 3.

For *J=2*, even in the non-sparse setting, the proposed method did not mistakenly shrink any necessary current to $g_j=0$, and it recovered both conductances close to the true values $\theta _{\textrm{true}}=[1.0, 1.5, 0, \dots , 0]^\top$. For *J=4, 8, 12,* and 16, even when we increase the sparsity level, the method successfully kept the truly necessary currents ($I_1$ and $I_2$) non-zero, while shrinking the unnecessary candidates ($I_3$ to $I_{16}$) to zero. Across all values of *J*, the proposed method correctly identified the necessary currents ($I_1$ and $I_2$) and suppressed unnecessary candidates. This demonstrates robust selective current estimation under varying sparsity levels.

Furthermore, we investigate the results of estimation for different numbers of calcium currents in true model. Here, we consider multiple cases where true model has no calcium currents (*K=0*), two calcium currents (*K=2*) and four calcium currents (*K=4*), where we have 16 candidate calcium currents *(J=16)*. For the case of *K=4*, the true model contained four active calcium currents ($g_1$, $g_2$, $g_9$, and $g_{15}$). Table 4 shows the estimated maximal conductances under varying numbers of *K*. When no calcium currents are present (*K=0*), the proposed method successfully excludes all candidate currents. When calcium currents are present (*K=2* and 4), only the necessary currents are retained while the others are suppressed. In the cases where the calcium currents are present, we find that the values of membrane conductances are estimated accurately. These results further demonstrate the robustness of the proposed method in selectively identifying relevant membrane currents and accurately capturing neuronal nonlinear dynamics.

**Table 3:** Estimated maximal conductances $g_j$ for candidate calcium currents under different numbers of candidates *J* from one-dimensional noisy membrane potential observations. The true model contains only $I_1$ and $I_2$, and the other candidates are unnecessary and should be shrunk to zero

*j*	True	*J=2*	*J=4*	*J=8*	*J=12*	*J=16*
1	1.0	0.986468	0.985769	0.985458	0.989670	0.987682
2	1.5	1.536441	1.534505	1.534822	1.536999	1.536629
3	0.0		0.000000	0.000000	0.000000	0.000000
4	0.0		0.000000	0.000000	0.000000	0.000000
5	0.0			0.000000	0.000000	0.000000
6	0.0			0.000000	0.000000	0.000000
7	0.0			0.000000	0.000000	0.000000
8	0.0			0.000000	0.000000	0.000000
9	0.0				0.000000	0.000000
10	0.0				0.000000	0.000000
11	0.0				0.000000	0.000000
12	0.0				0.000000	0.000000
13	0.0					0.000000
14	0.0					0.000000
15	0.0					0.000000
16	0.0					0.000000

**Table 4:** Estimated maximal conductances for candidate calcium currents under different numbers of calcium currents *K* included in true model

*j*	True (*K=0*)	Estimation (*K=0*)	True (*K=2*)	Estimation (*K=2*)	True (*K=4*)	Estimation (*K=4*)
1	0	0.000000	1.0	0.987682	1.0	1.000473
2	0	0.000000	1.5	1.536629	1.5	1.505061
3	0	0.000000	0	0.000000	0	0.000000
4	0	0.000000	0	0.000000	0	0.000000
5	0	0.000000	0	0.000000	0	0.000000
6	0	0.000000	0	0.000000	0	0.000000
7	0	0.000000	0	0.000000	0	0.000000
8	0	0.000000	0	0.000000	0	0.000000
9	0	0.000000	0	0.000000	2	1.996615
10	0	0.000000	0	0.000000	0	0.000000
11	0	0.000000	0	0.000000	0	0.000000
12	0	0.000000	0	0.000000	0	0.000000
13	0	0.000000	0	0.000000	0	0.000000
14	0	0.000000	0	0.000000	0	0.000000
15	0	0.000000	0	0.000000	4	4.001035
16	0	0.000000	0	0.000000	0	0.000000

## Conclusion

In this paper, we propose a method to jointly estimate latent variables and biophysical parameters using a sequential Monte Carlo method and the EM algorithm with sparse modeling. Using a conductance-based neuron model that incorporates both membrane potential and calcium dynamics, the proposed method estimates the multi-dimensional latent variables using a sequential Monte Carlo method and updates the electrical parameters by maximizing the *Q* function that reflects neuronal dynamics with sparse modeling. The proposed method enables joint estimation of the membrane potential, intracellular calcium dynamics, and sparse selection of the electrical parameters that govern the entire process.

From only membrane potential observations, we successfully estimated latent variables under one-dimensional observation settings by a sequential Monte Carlo method and achieved convergence of the estimated electrical properties toward their true values. Moreover, the sparse extension successfully suppressed inactive current components while retaining the truly contributing currents. These results demonstrate the effectiveness of the proposed estimation framework for neuronal dynamics as the utility of sparse estimation for robust current selection and parameter estimation in neuronal models.

## Data Availability

Simulation code can be made available by the corresponding author (T.O.) upon reasonable request.
